# Opioids, Neutral Endopeptidase, its Inhibitors and Cancer: Is There a Relationship among them?

**DOI:** 10.1007/s00005-014-0311-0

**Published:** 2014-09-06

**Authors:** Magdalena Mizerska-Dudka, Martyna Kandefer-Szerszeń

**Affiliations:** Division of Biology and Biotechnology, Department of Virology and Immunology, Institute of Microbiology and Biotechnology, Maria Curie-Sklodowska University, Lublin, Poland

**Keywords:** Opioid peptides, OGF, Colon cancer, Neutral endopeptidase, CD10

## Abstract

The role of endogenous animal opioids in the biology of cancer is widely recognized but poorly understood. This is, among others, because of the short half-life of these peptides, which are quickly inactivated by endopeptidases, e.g., neutral endopeptidase (NEP, CD10). It has been established that NEP is engaged in the modulation of the tumor microenvironment, among others that of colon cancer, by exerting influence on cell growth factors, the extracellular matrix and other biologically active substances. Although there are some discrepancies among the findings on the role of both opioids and NEP in cancer development, authors agree that their role seems to depend on the origin, stage and grade of tumor, and even on the method of examination. Moreover, recently, natural inhibitors of NEP, such as sialorphin, opiorphin and spinorphin have been detected. Their analgesic activity has been established. It is interesting to ask whether there is a relationship among opioid peptides, tumor-associated NEP and its inhibitors.

## Opioid Peptides and Cancer Cells

Opioids are a group of both naturally occurring and synthetically produced molecules, traditionally known by their analgesic activity.

Opium alkaloids derived from plants, for example, morphine, are commonly used as medicines and are regarded as the “gold standard” for relieving severe pain associated with cancer (Lutz and Kieffer 2013). Moreover, other than pain-suppressive activities of exogenous opioids have been widely examined. It has been observed that morphine exerts both tumor-growth promoting and growth inhibiting activities, by influencing the proliferation and migration of tumor cells and angiogenesis as well as by affecting the immune system (Gach et al. 2011; Harimaya et al. 2002; Schäfer and Mousa 2009; Tabellini et al. 2014).

In animals, naturally occurring opioid peptides are synthesized as prohormones. Most currently known endogenous opioids originate from three types of prohormones: proenkephalins, pro-opiomelanocortins and prodynorphins (Hughes et al. 1975; Simon et al. 1973; Terenius 1973). Despite their different activities in living organisms, these endogenous peptides possess common structural features, namely, an N-terminal enkephalin sequence in molecules comprised of 5–40 amino acid residues (Civelli and Douglass 1986; Thanawala et al. 2008). [Met^5^]-enkephalin and [Leu^5^]-enkephalin, the first endogenous peptides discovered, differed only at the C-terminal residues (Akil et al. 1984).

## The Presence and Activity of Opioid Peptides

Many studies have indicated that opioid peptides possessing an enkephalin sequence are widely spread in the human body, where they play a broad variety of functions. Among others, they act as neuromodulators and growth factors through interaction with µ, δ and κ opioid receptors (McDonald and Lambert 2005; McLaughlin and Zagon 2012). Neuromodulatory activities of endogenous opioid peptides are connected with inhibition of the release of bioactive compounds, e.g., dopamine; regulation of pain and motor activity; gastrointestinal motility and peristalsis; regulation of emotional behavior; appetite and thirst and many others (Bauvois and Dauzonne 2006; Bohlen and Dermietzel 2006; McLaughlin and Zagon 2012).

The presence of opioid peptide receptors and/or their agonists has been established not only in neurons but also in immune cells (granulocytes, monocytes/macrophages, lymphocytes, natural killer cells) (Iwaszkiewicz et al. 2013; Li et al. 2013; Schäfer and Mousa 2009) and tumor cells originating from colon cancer (CC), breast cancer, lung cancer, pancreatic cancer, thyroid cancer, endocrinal tumors, endometrial carcinoma, glioma and melanoma (Fichna and Janecka 2004).

## Opioid Growth Factor

At the end of the twentieth century, Zagon and his team proposed a hypothesis that opioid peptides also act as growth regulators (McLaughlin and Zagon 2012). This was related especially to [Met^5^]-enkephalin which was, accordingly, renamed as the opioid growth factor (OGF) (Zagon and McLaughlin 1987, 1988, 1989, 1991; Zagon et al. 1987, 1997, 1999b). OGF is a non-cytotoxic molecule produced in an autocrine and paracrine fashion. Extensive research has demonstrated that OGF and its receptor, OGFr, exert their activity through protein/RNA synthesis-dependent regulation of cell proliferation (Bisignani et al. 1999; Donahue et al. 2009; Hatzoglou et al. 2005; McLaughlin and Zagon 2012). Studies on normal and cancer cells have revealed that OGF arrests the cell cycle at phase G_1_/S through modulation or upregulation of cyclin-dependent inhibitory kinases p16 and p21. This results in the inhibition of DNA synthesis and subsequent arrest of receptor-dependent proliferation of non-malignant and cancer cells (Cheng et al. 2007, 2008, 2009; Donahue et al. 2009). The OGF and its receptor are engaged in the maintenance of homeostasis through regulation of cell proliferation, tissue (heart, corneal epithelium, astrocyte, mesenchymal cell) development and renewal, as well as wound healing and angiogenesis (Blebea et al. 2000; Isayama et al. 1991; McLaughlin 1996; McLaughlin and Zagon 2012; Zagon and McLaughlin 1987, 1988, 1991; Zagon et al. 2000, 2002). The inhibitory activity of the OGF-OGFr axis has also been assessed in vitro and in vivo against cancer cells derived from neuroblastoma, pancreatic adenocarcinoma, colon adenocarcinoma, head and neck squamous cell carcinoma, and renal, ovarian and breast tumors (Donahue et al. 2009; Hatzoglou et al. 2005; McLaughlin and Zagon 2012; McLaughlin et al. 1999a, b; Zagon et al. 1999a, 2009). On the other hand, there are some reports which indicate that opioid peptides (especially [Met^5^]-enkephalin) secreted by cancer cells, e.g., colon cancer, can suppress immune response to promote cancer progression and invasiveness (Ohmori et al. 2009). In the case of CC, a relationship has been found between the expression level of the opioid peptide and tumor-infiltrating T lymphocytes. In general, the number of lymphocytes T, bearing the δ opioid receptor, decreased along with increased Met^5^-enkephalin expression (Ohmori et al. 2009).

## Enzymes Degrading [Met^5^/Leu^5^]-Enkephalin Type Opioids

Opioid peptides are engaged in a wide range of processes, presenting both advantages and disadvantages to living organisms. Their characteristic feature is a short half-life associated with the rapid degradation of those peptides by endogenous enzymes (Janecka et al. 2008; Kreczko and Maćkiewicz 2011). Neutral endopeptidase (NEP, neprilysin, enkephalinase, CD10, EC 3.4.24.11, common acute lymphoblastic leukemia antigen) and aminopeptidase N (APN, CD13, EC 3.4.11.2) are two of the many enzymes involved in the degradation of endogenous opioid peptides (Janecka et al. 2008; Kreczko and Maćkiewicz 2011; Schreiter et al. 2012). These enzymes are widely distributed in living organisms and engaged in diverse physiological and pathological processes.

NEP and APN are membrane-bound, zinc-dependent metallopeptidases of the gluzincin family. *NEP* gene is located on human chromosome 3q21–27, whereas *APN* gene on chromosome 15q25-26 (Maguer-Satta et al. 2011; Noren et al. 1997). Both genes may be differentially expressed, in a tissue-specific manner (Carl-McGrath et al. 2006; Maguer-Satta et al. 2011). The *CD10* gene encodes a 90–110 kDa and *CD13* gene an approximately 150 kDa type II membrane protein (Carl-McGrath et al. 2006; Maguer-Satta et al. 2011; Noren et al. 1997). NEP cleaves peptide bonds on the amino side of hydrophobic residues and has also peptidyl-dipeptidase activity with some substrates (Rogues et al. 1993), whereas APN preferentially cleaves N-terminal unsubstituted neutral amino acids from proteins (Noren et al. 1997).

## Implications of NEP and APN for Health and Disease

Neutral endopeptidase and APN are widely distributed among different tissues and organs, where they play certain roles.

### Neutral Endopeptidase

In the central nervous system, NEP processes enkephalin, an opioid peptide liberated by neurons in response to pain and substance P (Rogues et al. 1993). NEP is known as an amyloid β-peptide-degrading enzyme. Its dysfunction leads to an accumulation of insoluble neurotoxic β amyloid peptide and neuronal death in Alzheimer’s disease (Iwata et al. 2001; Yasojima et al. 2001). Recently, it has been demonstrated that the neuroprotective activity of kynurenic acid is associated, at least partially, with the induction of the expression and/or activity of NEP in nerve cells (Klein et al. 2013). In the immune system, NEP is present on the surface of neutrophils. It regulates the activation of immunocompetent cells by degrading inflammatory peptides such as endothelin, bradykinin, atriopeptin and interleukin-1. It is known that NEP also processes somatostatin, neurokinin, cholecystokinin-8, angiotensin-I and-II, gastrin-related protein, calcitonin, calcitonin gene-related peptide and bombesin. NEP (CD10) has been used as a marker of stem cells in normal tissues. It is engaged in tissue morphogenesis and cell differentiation, among others in the lung and mammary gland. This enzyme is also implicated in the maturation of B cells (Carl-McGrath et al. 2006; Maguer-Satta et al. 2011).

Many previous studies have indicated that CD10 plays an important role in tumor progression (Carl-McGrath et al. 2006; Fujita et al. 2007). NEP (CD10) might be a very useful tool in the diagnosis and prognosis of B-lineage acute lymphoblastic leukemia and several carcinomas originating from kidney, lung, skin, pancreas, prostate, liver, breast, stomach, cervix and bladder. It has been detected that NEP can be up- or down-regulated in neoplastic cells. Moreover, it should be underlined that the expression level of NEP is dependent on the proliferation and differentiation status of tumor cells. NEP is implicated both indirectly and directly in the regulation of signaling pathways mediating cell migration, proliferation and survival. This indirect action results from proteolytic degradation or activation of bioactive peptides, growth factors and cytokines, which creates a microenvironment that facilitates tumor cell proliferation, invasion and metastasis. In addition to its function mediated through enzymatic activity, NEP regulates signaling pathways in a direct fashion. It acts as an immune receptor anchored in the cell membrane through GPI-complexes. CD10 is implicated in cell migration, cell proliferation and survival through focal adhesion kinase and PTEN/AKT signaling pathways. These functions of NEP have been extensively explored among others in prostate cancer, but not in CC (Carl-McGrath et al. 2006; Maguer-Satta et al. 2011; Sumitomo et al. 2000, 2001, 2004, 2005). The CD10 antisense S-oligodeoxynucleotide treatment of CD10-positive CC cell line, HT-29 resulted in inhibition of growth, invasion and colony formation (Luo et al. 2009). Further studies indicated that NEP contributes to liver metastasis of CC cells by degradation of the anti-tumoral peptide, Met^5^-enkephalin (Kuniyasu et al. 2010; Luo et al. 2009).

### Aminopeptidase N

The numerous studies on APN biological roles revealed that APN is involved in both physiological and pathological processes including cancers and inflammatory diseases. APN plays its functions through degradation of diverse bioactive peptides, e.g. vasoactive peptides, neuropeptides, chemotactic peptides and extracellular matrix (ECM) (Bauvois and Dauzonne 2006; Carl-McGrath et al. 2006). Moreover, APN is implicated in cell signaling pathways involving MAP kinases and Wnt-5a protooncogen (Lendeckel et al. 1998, 2000).

It was widely investigated that APN is engaged in regulation of cell growth and maturation (Bauvois and Dauzonne 2006). Similar to NEP, APN is considered as a marker of differentiation as it is expressed on stem cells and on leukocytes dependent on their stage of differentiation (Razak and Newland 1992a, b). Moreover, it is implicated in immune response mechanisms. APN is considered to regulate the secretion of proinflammatory and immunosuppressive cytokines (Lendeckel et al. 2003; Mishima et al. 2002). The important role APN plays in modulation of cell motility. It is executed not only by chemokines processing but also the degradation of ECM components. Taking into account the fact that many types of malignant cancers overexpressed APN, this function of peptidase seems to be especially important in tumor invasiveness and metastasis (Carl-McGrath et al. 2006; Fujii et al. 1996; Hashida et al. 2002; Saiki et al. 1993). Moreover, the ability of APN to EMC degradation implicates it in both physiological and pathological angiogenesis processes (Aozuka et al. 2004; Bhagwat et al. 2001; Fukasawa et al. 2006; Pasqualini et al. 2000; van Hensbergen et al. 2003). It was also demonstrated that APN acts as an adhesion molecule during angiogenic morphogenesis (Fukasawa et al. 2006).

## Colon Cancer

The development and growth of tumor cells result from the accumulation of mutations during carcinogenesis. These mutations lead to essential alterations in cell physiology that dictate malignant growth characterized by self-sufficiency with respect to growth signals, insensitivity to growth-inhibitory signals or sensitivity to weak growth signals not detected by normal cells; resistance to cell signals inducing death and elimination of defective cells; limitless replicative potential; and the ability to induce angiogenesis, evasion of the nearest tissues and metastasis (Hanahan and Weinberg 2000). The promotion of proliferation, inhibition of apoptosis, invasion and migration through tissues as well as the induction of angiogenesis during carcinogenesis are regulated by autocrine and paracrine growth factors, cytokines, hormones and signaling peptides. The availability of these extracellular signaling molecules is regulated through proteolysis, which may be mediated among others by cell membrane-bound peptidases, e.g. NEP and APN, expressed on the surface of tumor, stromal and endothelial cells. These multifunctional proteins are asymmetrically oriented in the cell membrane, with the catalytic site exposed to the extracellular surface. This enables them to release many growth factors and their receptors into the circulation, remodel the ECM as well as activate or inactivate the circulating signaling molecules through proteolysis (Carl-McGrath et al. 2006).

Colon carcinoma is one of the most frequent tumors in the world. The life expectancy in patients with the metastatic form of this tumor has been extended in the past decade, but the disease still remains incurable. The development of CC, which starts with changes in normal colonic epithelium through adenomatous polyps to metastatic cancer is dependent on and supported by the tumor microenvironment. The components of the tumor microenvironment include stroma cells, vasculature, nerves and the ECM with associated molecules. The essential part of stroma cells are tumor-associated fibroblasts, which produce a set of factors facilitating tumor growth and promoting angiogenesis. They also enable tumor invasion and metastasis, and produce ECM components with associated molecules (Colucci et al. 2005, 2008; Mayordomo et al. 2012; Peddareddigari et al. 2010). Additionally, transformed epithelial cells modulate the function of their microenvironment to facilitate their own growth, survival, invasion and metastasis (Peddareddigari et al. 2010). One of the most important growth factors expressed by both colon cancer tumor cells and tumor-associated fibroblasts, which regulates cancer development, is tumor growth factor (TGF)-β. In the case of CC, its function depends on the tumor stage. In early stages, TGF-β1 acts as a tumor suppressor, whereas in later stages it presents cancer-promoting activities (Paduch and Kandefer-Szerszeń 2009; Peddareddigari et al. 2010). TGF-β1 is secreted in a latent complex, covalently linked to ECM, from which it is released by proteases such as metalloproteases (McMahon et al. 1996).

### The Expression of NEP by CC Cells

The studies on NEP/CD10 in CC cells were concerned mainly on comparisons of the protein level in tissue samples of colon adenocarcinoma with non-neoplastic adjacent tissues, with respect to degrees of tumor differentiation, invasion and metastasis. These reports, however, are discrepant in some aspects. For example, de Oliveira et al. (2011) and Sato et al. (1996) have reported that the level of CD10 expression was higher in CC tissue samples than in non-neoplastic mucosa adjacent to the tumor, whereas Ogawa et al. (2002) have not found any expression of this marker in samples of normal tissue. Sato et al. (1996) and Fujimoto et al. (2005) have identified a higher expression level of CD10 in well or/and moderately differentiated adenocarcinoma tissue specimens in comparison with poorly differentiated ones. On the other hand, de Oliveira et al. (2011) and Oshima et al. (2007) have not observed any difference in the expression regarding histological differentiation. The lymphatic, vascular and/or perineural invasion of colorectal carcinoma has been found to be associated with CD10 expression by Fujimoto et al. (2005) and Yao et al. (2002), whereas de Oliveira et al. (2011) and Fujita et al. (2007) have not observed a significant relationship of this kind. Moreover, there are some controversies regarding the expression level of CD10 and metastasis. Fujimoto et al. (2005) and Yao et al. (2002) have identified a strong expression of this marker in patients with a higher incidence of liver metastases. On the other hand, de Oliveira et al. (2011) and Fujita et al. (2007) have not found any relationship between these two parameters. It should be underlined that Fujita et al. (2007) studied the expression of CD10 on the mRNA level not the CD10 protein level. It is possible that those discrepancies are caused by the fact that those authors examined tumors at different stages of development.

## Inhibitors of Enkephalin-Degrading Enzymes

Rapid degradation of peptide opioids is especially undesired when they exert a beneficial action, e.g., analgesic, anti-inflammatory and antitumoral. This has prompted scientists to search for natural or synthetic inhibitors of enkephalin-degrading enzymes and endomorphin analogs with an increased stability (Janecka et al. 2008; Kreczko and Maćkiewicz 2011; Rougeot et al. 2003; Schreiter et al. 2012; Wisner et al. 2006; Yamamoto et al. 2002).

Recently, three naturally occurring inhibitors of enkephalin-degrading enzymes have been discovered: sialorphin, opiorphin and spinorphin.

Sialorphin is an exocrine and endocrine signaling pentapeptide (QHNPR), synthesized mainly in the submandibular gland and prostate of adult rats in response to androgen steroids. To our knowledge, the occurence of this peptide was determined in milk and placenta, in addition to saliva and urine (Dufour et al. 2013; Rougeot 2004). The sialorphin gene, *VCSA1*, encodes the precursor prohormone, the submandibular rat_1_ protein (SMR_1_). It is processed also into other bioactive peptides, namely heptapeptide with the sequence TDIFEGG, named submandibular gland peptide-T and its C-terminal derivative, tripeptide FEG. The last mentioned above peptides are considered as regulators of inflammatory and allergic reactions in various animal models (Mathison et al. 2010; Morris et al. 2007). Sialorphin is released locally and systematically from SMR_1_ protein. It is also acutely secreted in response to stress (Rougeot et al. 2003). Rougeot et al. (2003) have provided evidence that sialorphin is a natural inhibitor of the cell surface NEP in several mammals models. This indicates the lack of species-specific activity, similar to TDIFEGG peptide (Morris et al. 2007). It has been demonstrated that this peptide acts as a competitive inhibitor of renal and spinal NEP, inhibiting the degradation of substance P and Met^5^-enkephalin and thus displaying analgesic activity (Rougeot et al. 2003). It has also been postulated that sialorphin could be involved in the regulation of systemic mineral ion homeostasis due to distribution of its peripheral target sites within tissues engaged in the regulation of ion capture and transport (Rougeot et al. 2003). Moreover, this inhibitor causes the enhancement of sexual behaviour and increased erectile function of male rats (Kos and Popik 2005; Messaoudi et al. 2004).

Opiorphin is a human pentapeptide (QRFSR) which was first discovered in saliva and characterized by an inhibitory activity against two enkephalin-degrading enzymes, NEP and APN (Wisner et al. 2006). Recently, the expression of opiorphin was additionally identified in male reproductive system, in mammary and lachrymal glands. This results in the occurence of peptide in semen, milk, tears, human plasma and even urine (Dufour et al. 2013). It is of interest that the secretion and concentration pattern of opiorphin is differentiated. It depends on gender and type of organ (Dufour et al. 2013). It was determined that opiorphin displays analgesic and antidepressant activity in in vivo animal models of pain and depression (Javelot et al. 2010; Popik et al. 2010; Rougeot et al. 2010; Tian et al. 2009; Yang et al. 2011). It is worth to be underlined that opiorphin is as effective as morphine on acute pain (Rougeot et al. 2010). Moreover, it is well documented that opiorphin demonstrated weaker or even no adverse side effects e.g. addiction or tolerance at active doses used in in vivo tests what is typical for morphine usage (Popik et al. 2010; Rougeot et al. 2010). It is proved that opiorphin displays these features by potentiating the activation µ- and δ-opioid pathways. This is resulted from inhibition of NEP- and/or APN-mediated degradation of endogenous enkephalins, similarly as in case of sialorphin (Javelot et al. 2010; Rougeot et al. 2003, 2010; Tian et al. 2009; Wisner et al. 2006).

Spinorphin, a bovine heptapeptide (LVVYPWT) belonging to the hemorphin family has been isolated from the spinal cord (Nishimura and Hazato 1993b). It presents inhibitory activity against enkephalin-degrading enzymes such as NEP, APN and dipeptidyl peptidase III (Nishimura and Hazato 1993a). This feature of spinorphin is a prerequisite for its analgesic and anti-inflammatory activity (Chen et al. 2000; Honda et al. 2001; Polosa et al. 1997; Thanawala et al. 2008; Yamamoto et al. 1997, 2002). The in vitro and in vivo studies indicated that this inhibitor acts as antagonist of *N*-formylpeptide receptor subtype FPR which resulted in blockage of fMet-Leu-Phe-induced stimulation of neutrophils (Liang et al. 2001; Yamamoto et al. 1997).

## Conclusion

It is well documented that endogenous and exogenously administered opioids are engaged in the regulation of stress and inflammation, wound healing, elimination of pathogen invasion and tumor growth. The findings regarding the effects of the action of opioids, especially concerned tumor development, are, however, contradictory. This issue has been widely reviewed by Gach et al. (2011). Some studies have pointed to the inhibitory action of endogenous opioid peptides on normal and tumor cell proliferation. Their immunosuppressive action has been regarded as beneficial in chronic inflammation. On the other hand, there are reports indicating that this feature of opioids is detrimental to tissue repair and tumor progression and metastasis. In spite of these discrepancies, there is some interest among researchers, in substances that inhibit the activity of enzymes degrading opioid peptides, among other NEP. The use of such inhibitors would allow to replace the administration of detrimental exogenous opioids, e.g., morphine, in patients with tumors. Although the role of NEP in tumor biology is well established, there are still no data on the inhibition of tumor-associated NEP by naturally occurring inhibitors, e.g., sialorphin, opiorphin, spinorphin or their derivatives. It is, therefore, interesting and worthwhile to examine whether natural inhibitors and their synthetic derivatives could modulate the activities of cancer cells expressing NEP such as, for example, colon cancer cells. The natural inhibitors of NEP are peptide molecules easily degraded by endogenous proteases. Numerous studies are carried out to increase their stability and bioactive availability or to improve their biological activity in relation to amino acid sequence and molecule structure (Bogeas et al. 2013; Jung et al. 2007; Kamysz et al. 2013a, b; Kotynia et al. 2010; Rosa et al. 2012). This may be advantageous to relieve the pain connected with cancer development or treatment and simultaneously to abrogate or diminish the development of pathological processes. It seems that the study of the opioid peptides-neutral endopeptidase-NEP inhibitors axis could be a step towards achieving this goal. *VCSA1* gene encoding the precursor of rat sialorphin and the *PROL1* gene encoding human opiorphin are members of the same gene family identified in rat, mouse and human. It was proved that the products of these genes do not present species specificity considering the interactions with target. It has also been found that the target of sialorphin, opiorphin and bovine spinorphin, namely NEP, is highly conserved among different mammalian species (≥93 % sequence homology in rat and human) (Rougeot et al. 2003; Wisner et al. 2006). Taking into account the considerations above, these peptide inhibitors constitute potentially therapeutic inhibitors of NEP.

## Abbreviations

AKT: Protein kinase B/AKT
APN: Aminopeptidase N
CALLA: Common acute lymphoblastic leukemia antigen
CC: Colon cancer
CKI: Cyclin dependent inhibitory kinases
ECM: Extracellular matrix
FAK: Focal adhesion kinase
GPI-complex: Glycosyl phosphatidyl inositol complex
MAP kinases: Mitogen-activated protein kinases
mRNA: Messenger RNA
NEP: Neutral endopeptidase
NK: Natural killer cells
OGF: Opioid growth factor
OGFr: Opioid growth factor receptor
*PROL1*: Proline rich, lacrimal 1
PTEN: Phosphatase and tensin homolog deleted on chromosome Ten
SGP-T: Submandibular gland peptide-T
SMR_1_: Submandibular rat1 protein
TGF-β1: Tumor growth factor β1
*VCSA1*: Variable coding sequence A1
